# Multiple-Clone Activation of Hypnozoites Is the Leading Cause of Relapse in *Plasmodium vivax* Infection

**DOI:** 10.1371/journal.pone.0049871

**Published:** 2012-11-21

**Authors:** Flávia Carolina F. de Araujo, Antônio Mauro de Rezende, Cor Jesus F. Fontes, Luzia Helena Carvalho, Cristiana F. Alves de Brito

**Affiliations:** 1 Laboratory of Malaria, Centro de Pesquisas René Rachou – Fiocruz Minas, Belo Horizonte, Minas Gerais, Brazil; 2 Laboratory of Cellular and Molecular Parasitology, Centro de Pesquisas René Rachou – Fiocruz Minas, Belo Horizonte, Minas Gerais, Brazil; 3 Hospital Universitário Júlio Muller, UFMT, Cuiabá, Mato Grasso, Brazil; Federal University of São Paulo, Brazil

## Abstract

**Background:**

*Plasmodium vivax* infection is characterized by a dormant hepatic stage, the hypnozoite that is activated at varying periods of time after clearance of the primary acute blood-stage, resulting in relapse. Differentiation between treatment failure and new infections requires characterization of initial infections, relapses, and clone multiplicity in vivax malaria infections.

**Methodology/Principal Findings:**

Parasite DNA obtained from primary/relapse paired blood samples of 30 patients with *P. vivax* infection in Brazil was analyzed using 10 molecular markers (8 microsatellites and MSP-1 blocks 2 and 10). Cloning of PCR products and genotyping was used to identify low-frequency clones of parasites. We demonstrated a high frequency of multiple-clone infections in both primary and relapse infections. Few alleles were identified per locus, but the combination of these alleles produced many haplotypes. Consequently, the majority of parasites involved in relapse showed haplotypes that were distinct from those of primary infections. *Plasmodium vivax* relapse was characterized by temporal variations in the predominant parasite clones.

**Conclusions/Significance:**

The high rate of low frequency alleles observed in both primary and relapse infections, along with temporal variation in the predominant alleles, might be the source of reported heterologous hypnozoite activation. Our findings complicate the concept of heterologous activation, suggesting the involvement of undetermined mechanisms based on host or environmental factors in the simultaneous activation of multiple clones of hypnozoites.

## Introduction

Malaria is a blood disease caused by protozoan parasites. The source of human infection is mainly *Plasmodium falciparum* and *Plasmodium vivax* [1]. Approximately 225 million malaria cases and 780 000 deaths occurred in 2009, and 40% of the world population lives at risk of infection [2], [3]. *Plasmodium vivax* is the most widespread human malaria parasite and is the main source of malaria morbidity outside Africa [4]. In Brazil, roughly 350 000 cases of the disease are registered each year, 99% of which are in the Amazon region, and mainly *P. vivax* infections [5].

*Plasmodium vivax* is characterized by persistence of dormant parasite forms, the hypnozoites, in the liver for varying periods of time after clearance of the primary acute blood-stage. In general, while parasites from tropical zones, such as the Chesson strain, exhibit a short latent period before frequent episodes of relapse; parasites from temperate zones, such as the St Elizabeth strain, show a long latent period succeeded by few relapses [6]. In some areas, for example India, a mixed relapse pattern has been described [7]. These findings suggest regulation of relapse pattern, perhaps based on genetic programming of the parasites. Although the mechanisms that control relapses and determine their periodicity are largely unknown, many factors seem to contribute to both the timing and number of relapses. Previous studies have demonstrated that the number of sporozoites inoculated by the anopheline mosquito is a relevant factor [8], [9]. Other factors suggested in hypnozoite activation are (i) a previous *P. falciparum* infection [10]; (ii) the influence of vectors – occurrence of mosquito bites and anopheline species involved in these bites [6], [11]; (iii) systemic *P. vivax* febrile illness [12]; (iv) host immune response [13], [14]; (v) drug treatments [15], [16]; and (vi) regional variations, e.g. disease seasonality, latitude, altitude [17]–[19].

Elucidation of the source of recurrent infections is a challenge, since they can result from the asexual blood-stage re-emerging after treatment (recrudescence), from new infections, or from relapses caused by hypnozoite activation. In endemic locations, the probability of relapse varies from 20 to 80% [20]; with a current average of around 30% in tropical areas [6], [21], [22]. Thus, relapses have important implications for the control of *P. vivax* and also for the evaluation of drug treatment efficacy [23].

The molecular characterization of parasites of primary and recurrent infections is a crucial tool for adequately defining the epidemiology of relapse. Molecular markers have been used to genotype paired parasite DNA samples from primary episodes and relapses, and these have shown genetically similar or homologous parasites [24]–[26]. More recently, a predominance of heterologous parasites (parasites genetically different) has been demonstrated in relapses [21], [27]–[29]. The latter studies were based on microsatellite analyses, which are highly polymorphic and, in general, are not under selective pressure.

The genotyping of microsatellites has revealed a high frequency of multiple-clone *P. vivax*, i.e. individuals harboring more than one genetically distinct parasite, in areas with different levels of transmission [30]–[32]. Therefore, relapses may originate from activation of parasite clones identical to those of the primary infections (homologous parasites) or genetically distinct clones (heterologous parasites), making the task of identification of the relapse source difficult. In this study, we hypothesized that the multiple-clone infections usually described for *P. vivax* infections should also be frequent in relapses. To determine if this is true we carried out analysis of the genetic diversity of parasite DNA samples obtained from paired primary/relapse blood samples of *P. vivax*-infected patients who received antimalarial treatment (chloroquine plus primaquine) and were not subsequently exposed to *P. vivax* re-infection (to exclude novel infections).

## Materials and Methods

### Study Samples and Area

Parasite DNA samples were selected from a cryopreserved bank and kept at the Laboratory of Malaria at the Centro de Pesquisas Rene Rachou - Fiocruz, Belo Horizonte, MG. The following criteria were used to select 30 patients for primary/relapse paired DNA sampling: (i) an interval between the first acute episode and recurrence of 30 days to 12 months; (ii) *P. vivax* patients who were not re-exposed to malaria transmission during the interval between infection episodes; (iii) pregnant women were excluded; (iv) absence of other *Plasmodium* infection; and (v) a minimum age of 14 years. Thirty *P. vivax-*infected patients were selected (from 14–63 years, average age 37 years) whose malaria diagnosis and treatment were conducted at the Hospital Universitário Júlio Muller, UFMT, Cuiabá, MT, in the years 2001 to 2009 (Table S1). All patients were treated according to contemporary therapy guidelines of the Ministry of Health of Brazil (MS/SVS) with chloroquine (25 mg/kg/day for 3 days) and primaquine (0.5 mg/kg/day for 7 days) [33]. The patients provided written consent to store and analyze their blood samples in accordance with guidelines for human research as specified by the Brazilian National Council of Health (Resolution 196/96). This study was approved by the ethical committee of the Centro de Pesquisas René Rachou (Fiocruz): protocol numbers 05/2006 and 05/2010. Written consent was obtained from parents or guardians on the behalf of the minors participating in the study, as approved by the institutional ethics committee. Primary infection was defined by microscopic diagnosis of *P. vivax* at first admission of the individual to the hospital. Seven of the 30 patients reported it as their first malaria infection of life. Since the hospital is located in a non-endemic area for malaria, the locations of infection acquisition were widely dispersed in the Amazon area, at an average distance of 1,205 km from the hospital. The interval between relapse and primary infection or last acute malaria episode (for 2^nd^ and 3^th^ relapses) ranged from 31 to 185 days and was mainly concentrated in a period of one to three months (average 2.3 months) (Figure S1).

### DNA Extraction and PCR Amplification

DNA was extracted from whole blood samples using a genomic DNA purification kit (Puregene®, Gentra Systems, Minneapolis, MN, USA) or from filter paper using the QIAamp DNA Blood Mini Kit (Qiagen, Hilden, Germany) according to the manufacturer’s protocols. Eight loci of microsatellites (MS01, MS02, MS04, MS05, MS06, MS07, MS08, and MS11) and two loci of MSP1 (block 2 and 10) were amplified using specific primers and conditions described by Rezende et al. [34] and Koepfli et al. [35]. The 8 microsatellites selected for this study were highly polymorphic and composed of di-, tri- and tetranucleotide repeat units [34]. All markers are specific to *P. vivax* genome. Polymerase Chain Reactions (PCR) were performed using a gradient thermocycler (Eppendorf, Hamburg, Germany). The melting temperatures and magnesium concentration ranged from 50 to 60°C and from 0.75 to 1.50 mM, respectively [34]. A reaction volume of 20 µl containing 20 pmol of each primer (forward and reverse), 0.125 mM of dNTP, 1× buffer, and 1 U platinum Taq DNA polymerase (Invitrogen, Life Technologies Corporation, Carlsbad, CA, USA) was used. The cycling parameters were set to: 1 cycle at 94°C for 2 min, 40 cycles of 94°C for 30s, 50–60°C for 20s, and 72°C for 30s; and a final cycle at 72°C for 2 min. To assess the amplification, products were visualized in agarose gels stained with ethidium bromide (0.5 µg/ml).

### Microsatellites and MSP1 Genotyping

Amplified PCR products using the forward primers labeled with fluorescein were separated and differentiated using capillary electrophoresis in the automatic DNA sequencer MegaBACE (Amersham Biosciences, GE Life Sciences, Buckinghamshire, England). Their lengths and relative abundance (peak heights in electropherogram) were determined using MegaBACE Fragment Profiler, version 1.2, software, by reference to internal size standards (MegaBACE ET550-R). We measured allele frequencies using the predominant allele at each locus per sample; non-predominant alleles were recorded and used to estimate multiplicity of infection, since all markers are single-copy loci, and blood-stage malaria parasites are haploid. The highest peak in the electropherogram was defined as the predominant allele, and other peaks that reached the minimum level for detection were defined as rare or low-frequent alleles in a multiple-clone infection. The detectable cut-off value for peak height was set to 150 arbitrary fluorescence units (rFU).

### PCR Products Cloning

To determine the presence of low-frequency alleles in multiple infections, 32 PCR products from amplification of four primary/relapse paired DNA samples from infected patients were selected for cloning using two arbitrarily chosen markers. From those samples, the PCR products were cloned onto a pGEM-T Vector (Promega, Madison, USA), according to the manufacturer’s protocol. Recombinant vectors were used to transform *Escherichia coli* Top10 strain using thermal shock [36], and the cells were plated in LB agar supplemented with ampicillin (50 µg/ml). Up to 26 colonies (mean 10) of each cloned product were selected for mini-prep plasmid extraction using the Wizard® *Plus* SV Minipreps DNA Purification System (Promega). The obtained DNA was measured in a NanoDrop spectrophotometer (Thermo Scientific, Waltham, MA, USA) and used for parasite genotyping using the same two molecular markers. To check for multiple infections and possible slippage of the polymerase during amplification of the microsatellite, the size of the amplicons obtained from recombinant plasmids containing the cloned PCR products was compared with those from the original amplicons.

**Figure 1:**
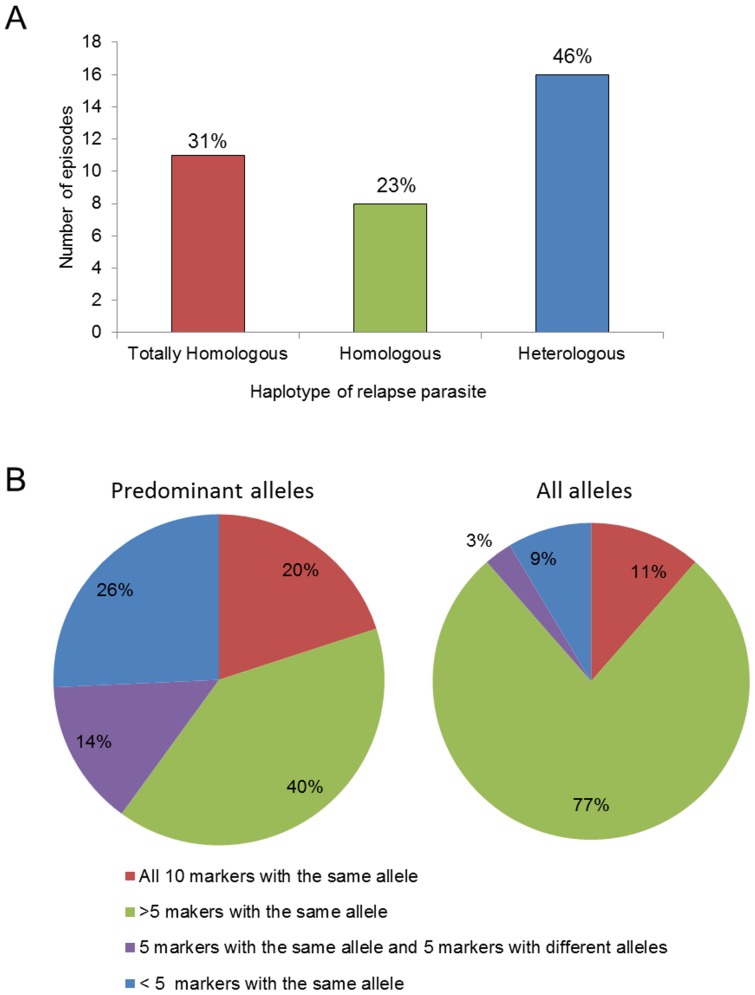
Genotyping of *P. vivax* primary/relapse paired parasites from 30 patients using 10 molecular markers. (A) Haplotype derived from predominant allele of each marker. Totally homologous – parasites showing all markers with the same allele; Homologous or Related – parasites with 8 to 9 markers with the same allele; Heterologous – parasites showing less than 8 markers with the same allele (according to Orjuela-Sánchez et al. [21]). In patients with more than one relapse episodes, relapse parasites were compared with the previous acute malaria episode. (B) Percent of acute malaria episodes showing different amounts of markers with the same alleles, taking into account only the predominant allele from each marker (left) or all alleles, predominant and rare from each marker (right).

### Statistical Analysis

We calculated the gene diversity (expected heterozygosity, *H*_E_), defined as the probability that a pair of alleles randomly obtained from the population differ, using Arlequin 3.0 software [37]. Genetic diversity of primary and relapse parasites was compared using analysis of molecular variance - AMOVA [38].

**Figure 2:**
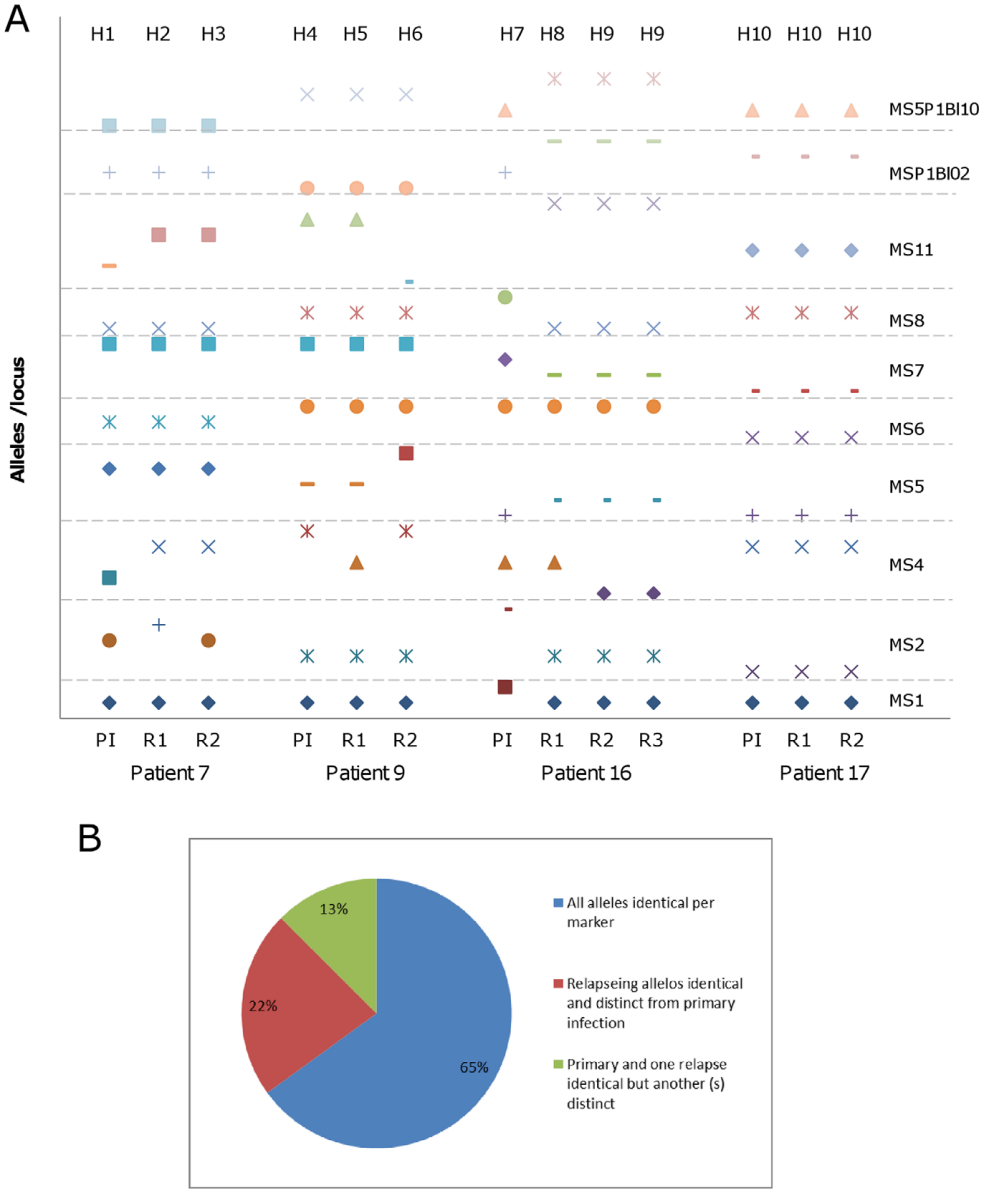
Temporal variation of the predominant alleles. (A) Comparison of predominant alleles among primary infection (PI), first relapse (R1), second relapse (R2), and third relapse (R3) from four *P. vivax*-infected patients genotyped using 10 molecular markers. Alleles are represented by different forms for each marker (indicated on the right side) and delimited by dotted lines. MS – microsatellite numbered according to Rezende et al. [36], MSP1bl2 and MSP1bl10– merozoite surface antigen 1 blocks 2 and 10, respectively. Haplotypes are indicated at the top of the Figure. (B) Frequencies of markers showing the same or distinct alleles at different times of blood collection for these four patients.

**Table 1:** Allele frequencies (%) and genetic variability of each molecular marker from *Plasmodium vivax* isolates.

	Percentage of episodes (%)									
Alleles	MS1	MS2	MS4	MS5	MS6	MS7	MS8	MS11	MSP1bl2	MSP1bl10
1	11	1	11	5	5	3	2	2	1	17
2	9	5	6	28	3	34	3	2	12	5
3	71	3	20	11	9	25	28	3	23	9
4	3	1	1	6	58	5	31	3	8	14
5	6	54	8	31	3	17	16	2	29	15
6		5	1	8	1	12	5	3	8	1
7		8	5	2	1	3	16	8	18	14
8		6	1	5	6	1		3		3
9		15	17	2	1			2		1
10		1	12	3	3			2		15
11			5		5			2		3
12			12		3			11		1
13								2		
14								6		
15								2		
16								2		
17								3		
18								2		
19								3		
20								3		
21								5		
22								2		
23								3		
24								3		
25								3		
26								3		
27								2		
28								8		
29								3		
30								2		
31								3		
Total	5	10	12	10	12	8	7	31	7	12
*H*_E_	0.482	0.681	0.888	0.808	0.647	0.789	0.783	0.969	0.813	0.886

MS: microsatellites [36]; MSP1bl2 and MSP1bl10: merozoite surface protein 1 block 2 and 10; *H*_E_: Expected heterozygosity; Total: number of distinct alleles for each marker. Alleles were numbered from smallest to the highest, each marker showed distinct sizes for the alleles, please see Supplementary Table S2 for the sizes of each allele.

## Results

Of the 30 patients studied, 26 had a single recurrence of parasitemia and 4 had two or more recurrent infections, for a total of 35 recurrences over the follow up period (Figure S1). *Plasmodium vivax* DNA from 65 acute infections showed high haplotype variability, with 55 haplotypes identified using a panel of 10 markers (haplotype diversity = 0.9957±0.0037) (Table S2). The expected heterozygosity varied from 0.48 to 0.96 among the loci (average 0.77±0.13).

**Figure 3:**
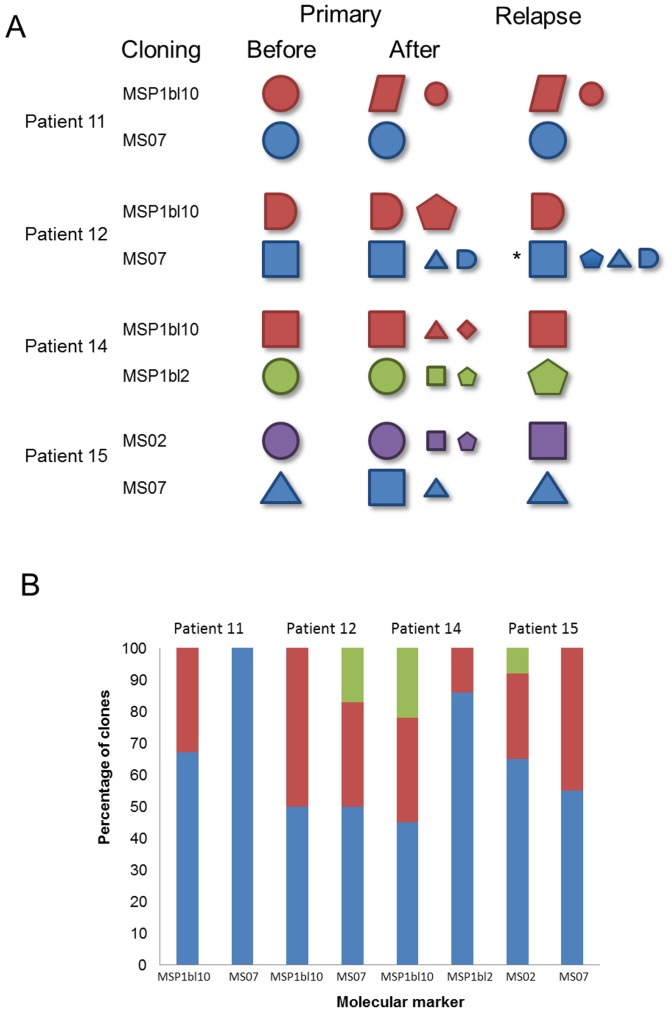
Genotypic profile before and after PCR cloning. PCR products from primary infection samples amplified using two randomly chosen molecular markers of four patients were cloned, and up to 26 colonies (mean of 11 colonies) were genotyped. (A) Each form represents an allele, size indicates predominant (larger) or rare alleles (smaller); color represents alleles of a distinct marker: MSP1bl10– red; MS07– blue; MSP1bl2– green; MS02– purple. The presence of two or more forms characterizes a multiple-clone infection. Before cloning the predominant allele was identified as the heighest peak in genotyping and the rare allele as the peak with one-third of the predominant peak height. After cloning the frequencies were inferred by the number of bacteria clones. The only relapse sample also cloned is indicated by an asterisk. (B) Frequency of each allele in primary infections after cloning measured by the percent of bacterial colonies genotyped with each allele (represented by different colors).

Considering the predominant allele in the haplotypes, analysis of primary/relapse paired DNA samples showed the highest proportion of parasites with heterologous haplotypes (46%), i.e. different alleles in relapse compared to primary infection were harbored in more than 2 of 10 markers (Fig. 1A), as previously reported by Orjuela-Sánchez et al. [21]. No parasite showed an entirely different haplotype in primary and relapse episodes. Fifty-four percent of the relapse parasites carried haplotypes related to or totally homologous to the primary infection. Expected heterozygosity was not significantly different in parasites of primary infections from those of relapse episodes (primary: *H*_E_ = 0.779±0.129; first relapse: *H*_E_ = 0.798±0.115; second relapse: *H*_E_ = 0.810±0.197; AMOVA test *p* = 0.7116).

**Figure 4:**
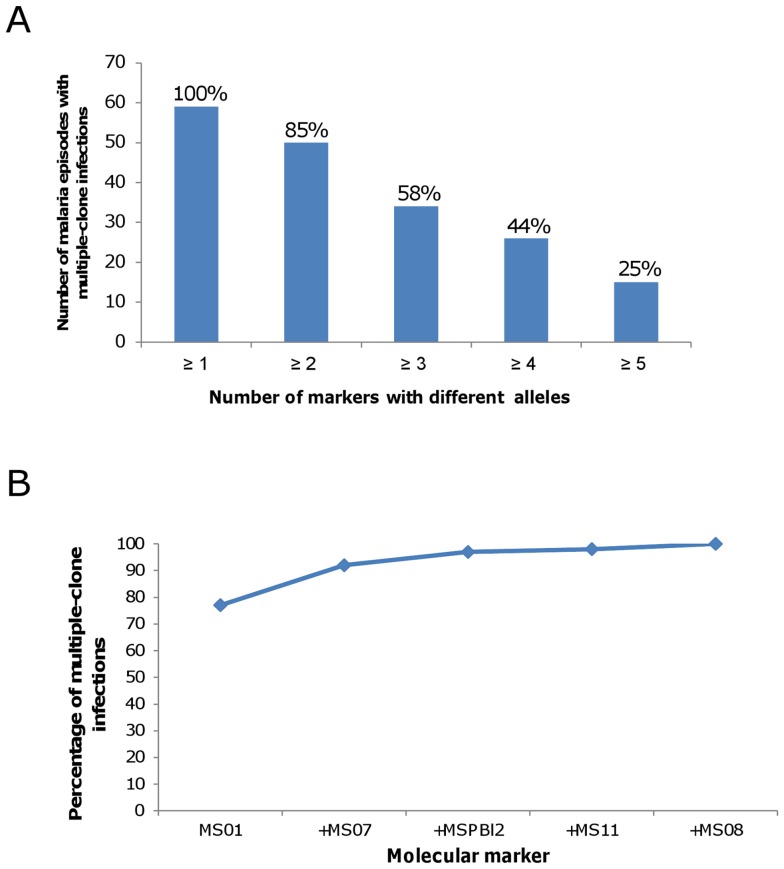
Detection of multiple-clone *P. vivax* infections using a panel of 10 markers. (A) Number and percent of malaria episodes showing multiple-clone infections detected by different numbers of markers. (B) Minimum number of markers able to detect all multiple clone infections was five: MS01 (77%), MS01+ MS07 (92%), MS01+ MS07+ MSPBl2 (97%), MS01+ MS07+ MSPBl2+ MS11 (98%), MS01+ MS07+ MSPBl2+ MS11+ MS08 (100%).

Few predominant alleles were detected by each marker, in general ranging from 5 to 12; one marker (MS11) identified 31 alleles (Table 1 and Table S2). The number of all alleles (predominant and rare) detected in the primary and relapse episodes was low. The average number of alleles was 9.2±2.3 and 10.8±2.7 for the predominant and all alleles, respectively, without including MS11 data, and 11.4±7.3 and 13.4±8.7 with MS11 data. A high frequency of predominant alleles of the same size was observed in the majority of markers. For all alleles (predominant and rare), this frequency increased from 40 to 77% (Fig. 1B). This finding reflects variation in how well the technique could detect low frequency alleles among the 10 markers used and suggested a significant temporal variability in predominant and rare alleles during the course of infection. In four patients with multiple recurrent infections, it was possible to confirm this temporal variation in predominant alleles (Fig. 2A). In these patients, each marker identified between two and 6 predominant alleles. Usually, those markers identified the same predominant allele in all consecutive samples from each patient in multiple malaria episodes (25 of 40, 65%) (Fig. 2B). Nine of the 15 remaining alleles were identical during relapses, but distinct from the primary infection. The combination of these alleles resulted in 10 haplotypes (H1 to H10). Only one patient harbored the same haplotype in all episodes (Patient 17; Fig. 2).

To clarify whether the detection of distinct alleles in relapses was due to their presence, although at low levels, in the preceding acute episodes, PCR products of two randomly selected markers from the primary infection of four patients were cloned, and up to 26 bacterial colonies (mean 11) were genotyped per product (Fig. 3). The majority of cloned samples were found to include more than one allele, representing a multiple-clone infection in both initial and recurrent infections. Up to 4 distinct alleles per marker were identified in a single patient (Fig. 3A). The frequency of colonies with the same allele ranged from 8 to 100% (Fig. 3B). These data strongly support the hypothesis that those distinct alleles detected in the recurrent infections correspond to undetected low frequency clones present in the primary infection.

To further assess whether rare alleles were not identified in the primary infection because of low levels of fluorescence, we re-analyzed the original electropherogram peaks. Predominant peaks could have different heights, while the rare ones may have been of similar height (Figure S2). As an example of genotyping, in one patient the higher peak corresponding to the predominant allele had 455 rFU, and the rare allele had 153 rFU; that is, around 33% the predominant peak height. Genotyping the same marker for a second patient showed a higher peak of 4665 rFU and a lower peak with similar intensity of fluorescence as before (188 rFU); that is 4% of the predominant peak height. Reinforcing these data, the presence of rare alleles was further confirmed by cloning (data not shown). Thus, by properly adjusting the cut-off (≥150 rFU), the molecular markers detected a high frequency of multiple-clone infections. In 59 of 65 samples (91%) multiplicity of infection was detected by at least one marker (Fig. 4A). Based on a minimum of two markers, the percent of multiple infections detected decreased to 77%, and, with a minimum of three markers, it was 52% (Fig. 4A). We also observed a wide variation among the molecular markers in their ability to identify multiple infections, with two microsatellite markers (MS01 and MS07) accounting for most of the detection (Fig. 4B). Although five markers were able to detect all multiple-clone infections, the results suggested that few markers could be used to detect the majority of multiple-clone infections.

**Figure 5:**
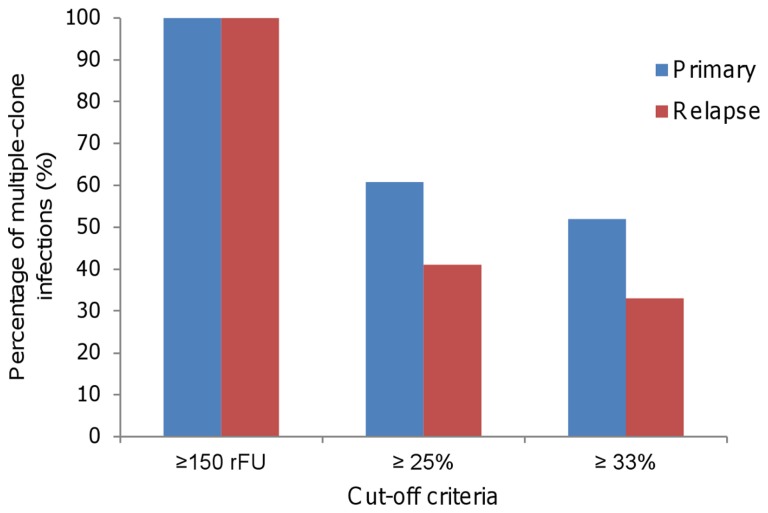
Percent of multiple-clone infections using different cut-off criteria. The detection of rare alleles in the genotyping was based on three cut-off criteria: ≥150 rFU (here); peaks with more than one quarter [46]; or with more than one third of the predominant peak height [47].

## Discussion

The activation of heterologous hypnozoites seems to be the most common cause of malaria recurrences [27], [29], [31], [39]. The results presented here reinforce previous studies, showing that the majority of relapse episodes were caused by a parasite population distinct from the primary infection. The novel finding of our study was the identification of high multiplicity not only in primary infections but also in relapses. These findings add complexity to the concept of heterologous activation, since they suggest that the allele present in relapses might also be present in the primary infection as a rare allele. Furthermore, the predominant parasite in the primary infection might not be predominant in the relapse, which shows that the frequency of circulating parasite clones alters considerably in *P. vivax* recurrences. Consequently, activation in relapse might be from homologous or heterologous hypnozoites or both (Table 1). Koepfli et al. [40] similarly reported temporal variation in the predominant allele during the course of *P. vivax* primary infection. In a study of *P. vivax* patients in the Peruvian Amazon, Van Den Eede et al. [39] reinforced this hypothesis of high turnover of parasite genotypes in recurrences. These findings point to the need for further studies to analyze multiple-clone infection during *P. vivax* recurrence, specifically with respect to primaquine resistance.

Numerous studies have addressed the mechanisms of hypnozoite activation. Recently, data from a study of infants in Thailand demonstrated that the first *P. vivax* relapse in life is usually caused by genetically homologous parasites. The authors suggest that this reflects the lack of previously acquired hypnozoites to be activated [41]. Accordingly, in the present study, five of seven patients showing a first malaria episode of their life exhibited a single-clone infection, with homologous parasites in recurrences. Notwithstanding, it is important to consider that malaria infection can be induced by the inoculation of more than one clone of sporozoites (multiple-clone infection) and, as hypothesized here, more than one clone of hypnozoites can remain dormant until some are activated. Consequently, it is not possible to determine if the heterologous hypnozoites are the first activated, which would explain their prevalence in relapses, or if the prevalence is related to the number of heterologous/homologous dormant clones that could be activated [27]. Since, in the current study, most of the initial infections did not correspond to the first sporozoite inoculation of life, previous infections could also be a source of heterologous hypnozoites.

Our aim was to improve sensitivity of detecting multiple-clone infections. The approach used to identify rare alleles was based on analysis of the electropherogram using a low cut-off level (≥150 rFU). By cloning PCR products, it was possible to confirm the specificity of this strategy and identify high levels of multiple-clone infections. By using a more common criterion to detect rare peaks, based on quantification of peak heights [42], [43], multiple-clone infection was confirmed in our primary/relapse samples (Fig. 5). However, this criterion has limitations, depending on the height of the predominant peak. Moreover, as multiplicity of infection has been demonstrated for different *P. vivax* populations [31], [32], [44]–[47], we believe that it is a common phenomenon of relapse parasites, that is not yet identified due to the low sensitivity of previous protocols. In order to reduce the artifact in genotyping, we used several strategies: repeat genotyping using different PCR products from the same patient to avoid dropout; confirming rare alleles using cloning before genotyping to detect null or silent alleles; and reduced stutter peaks (peaks closer to, or attached to, the main peak result from DNA slippage during PCR) in PCR standardization or discarding them from the analysis. In conclusion, our approach is useful to detect rare clones, but should be used with caution to avoid an overestimation of multiple-clone infections.

Our results showed high haplotype variability and multiplicity of clones in parasites from relapsed patients. These findings complicate the concept of heterologous activation, suggesting the involvement of undetermined mechanisms based on host or environmental factors in the simultaneous activation of multiple clones of hypnozoites. This study provided new insights into molecular biology of malaria relapse that must to be considered in control strategies for the disease.

## Supporting Information

Figure S1 **Interval between primary infection and relapse infections.** Time interval in months between primary and relapse per individual (A) and frequency of episodes at differing intervals (B). Repeated individual number represents a second relapse episode (red bar) and a third relapse episode (green bar) in the same patient. Interval of second and third relapses was measured in relation to previous acute malaria episode. Primary infections corresponding to the first malaria infection of the individual’s life are denoted by an asterisk (above the bars).
(TIFF)

Figure S2 **Genotyping of two DNA samples with distinct profiles for the same marker (MSP1Bl02).** (A) Multiple infection detected in which the minor peak showed around 33% of the fluorescence level (rFu) of the predominant peak (blue peaks). (B) Multiple infection detected in which the lower peak (rare allele) showed 4% of fluorescence level of the predominant peak. Fragment sizes are represented on the × axis of the graph. Red peaks represents the molecular marker used (MegaBACE™ ET550-R).
(TIFF)

Table S1 **Description of patient characteristics.**
(DOCX)

Table S2 **Characteristics of predominant alleles from genotyping of 10 molecular markers of** ***Plasmodium vivax*** **infected patients.**
(DOCX)
