# Supplementary figures and images for: Multiple-Clone Activation of Hypnozoites Is the Leading Cause of Relapse in Plasmodium vivax Infection
Source: PLoS One. 2012 Nov 21;7(11):e49871. doi: 10.1371/journal.pone.0049871 (PMC3503861; doi:10.1371/journal.pone.0049871)

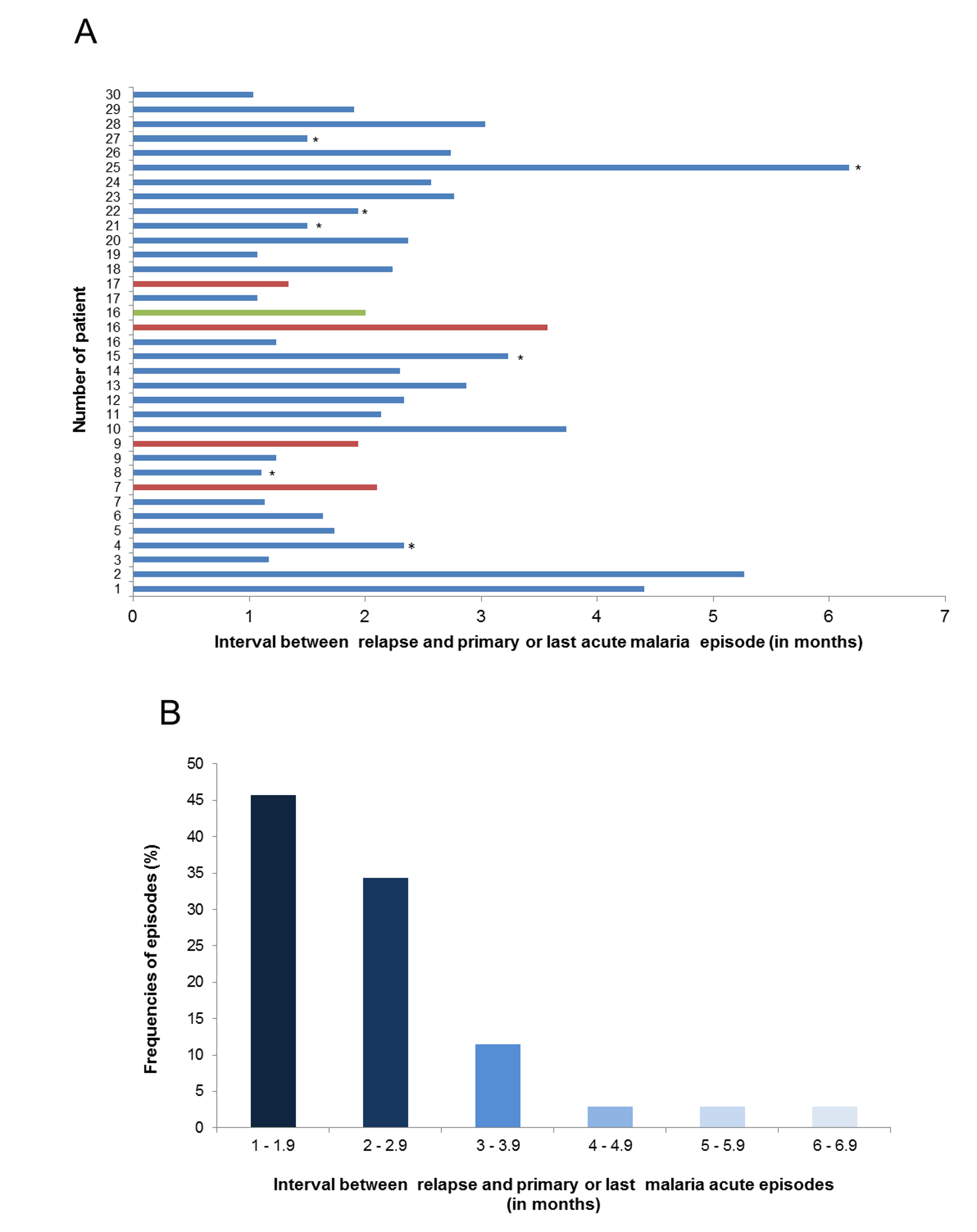

Supplement: Figure S1 — Interval between primary infection and relapse infections. Time interval in months between primary and relapse per individual (A) and frequency of episodes at differing intervals (B). Repeated individual number represents a second relapse episode (red bar) and a third relapse episode (green bar) in the same patient. Interval of second and third relapses was measured in relation to previous acute malaria episode. Primary infections corresponding to the first malaria infection of the individual’s life are denoted by an asterisk (above the bars). (TIFF) [file pone.0049871.s001.tiff]

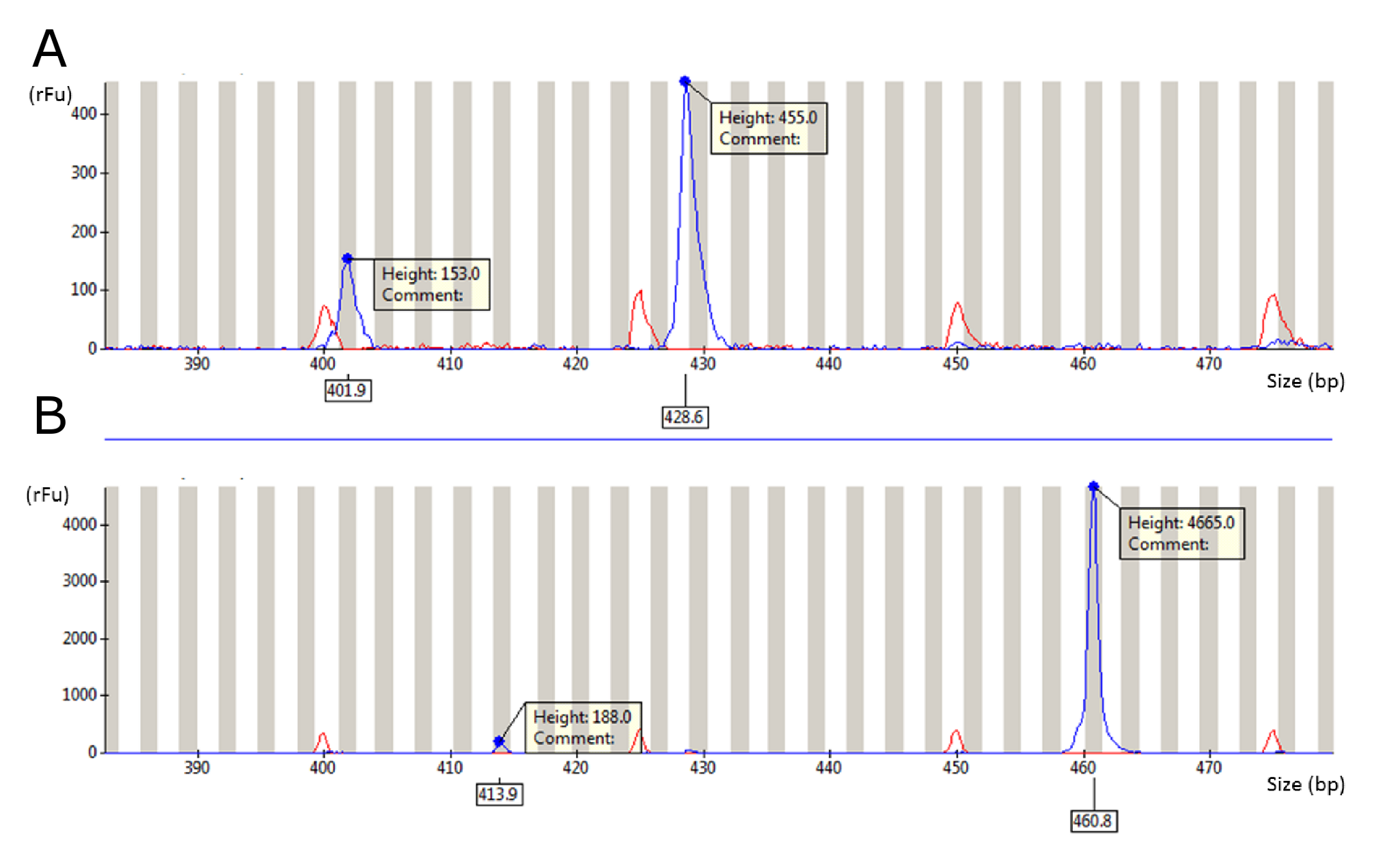

Supplement: Figure S2 — Genotyping of two DNA samples with distinct profiles for the same marker (MSP1Bl02). (A) Multiple infection detected in which the minor peak showed around 33% of the fluorescence level (rFu) of the predominant peak (blue peaks). (B) Multiple infection detected in which the lower peak (rare allele) showed 4% of fluorescence level of the predominant peak. Fragment sizes are represented on the × axis of the graph. Red peaks represents the molecular marker used (MegaBACE™ ET550-R). (TIFF) [file pone.0049871.s002.tiff]
