# Supplementary material for: Multiple-Clone Activation of Hypnozoites Is the Leading Cause of Relapse in Plasmodium vivax Infection
Source: PLoS One. 2012 Nov 21;7(11):e49871. doi: 10.1371/journal.pone.0049871 (PMC3503861; doi:10.1371/journal.pone.0049871)
Supplement: Table S1 — Description of patient characteristics. (DOCX) [file pone.0049871.s003.docx]

| **Table S1. Patient characteristics.** | | | | | |
| --- | --- | --- | --- | --- | --- |
| Patient number | Malaria episode | Data blood collection (Day/month/year) | Parasitemia (infected RBC/μL) | Age | Contagion localities |
| 1 | Primary | 12/03/2007 | 2412 | 31 | Itaituba/AM |
|  | Relapse | 22/07/2007 | 686 |  |  |
| 2 | Primary | 06/04/2006 | 3635 | 64 | Espigão D'Oeste/RO |
|  | Relapse | 11/09/2006 | 5180 |  |  |
| 3 | Primary | 04/07/2005 | 684 | 16 | São João Del Rey/MT |
|  | Relapse | 08/08/2005 | 425 |  |  |
| 4* | Primary | 13/10/2005 | 49 | 54 | Rio Branco/AC |
|  | Relapse | 22/12/2005 | 415 |  |  |
| 5 | Primary | 23/06/2009 | 700 | 47 | Ariquemes/RO |
|  | Relapse | 15/08/2009 | 700 |  |  |
| 6 | Primary | 22/02/2007 | 3158 | 25 | Humaitá/AM |
|  | Relapse | 11/04/2007 | 1343 |  |  |
| 7 | Primary | 02/08/2006 | 5836 | 23 | Ariquemes/RO |
|  | Relapse | 05/09/2006 | 519 |  |  |
|  | Relapse | 08/11/2006 | NA |  |  |
| 8* | Primary | 16/02/2007 | 2145 | 29 | Santo Antônio do Leverger/MT |
|  | Relapse | 21/03/2007 | 1786 |  |  |
| 9 | Primary | 19/02/2005 | 895 | 31 | Manicoré/AM |
|  | Relapse | 28/03/2005 | 200 |  |  |
|  | Relapse | 25/05/2005 | 1460 |  |  |
| 10 | Primary | 18/03/2005 | 1630 | 37 | Novo Progresso/PA |
|  | Relapse | 08/07/2005 | 719 |  |  |
| 11 | Primary | 21/12/2006 | 1618 | 33 | British guyana |
|  | Relapse | 23/02/2007 | 2910 |  |  |
| 12 | Primary | 03/10/2006 | 418 | 14 | Pontes e Lacerda/MT |
|  | Relapse | 12/12/2006 | 32 |  |  |
| 13 | Primary | 06/07/2005 | 2418 | 48 | Machadinho D'Oeste/RO |
|  | Relapse | 30/09/2005 | 1256 |  |  |
| 14 | Primary | 21/07/2005 | NA | 36 | Porto Velho/RO |
|  | Relapse | 28/09/2005 | 1344 |  |  |
| 15* | Primary | 05/09/2006 | 3087 | 41 | Colniza/MT |
|  | Relapse | 11/12/2006 | 8238 |  |  |
| 16 | Primary | 12/09/2005 | 2610 | 61 | Machadinho D'Oeste/RO |
|  | Relapse | 19/10/2005 | 620 |  |  |
|  | Relapse | 03/02/2006 | 2260 |  |  |
|  | Relapse | 30/03/2006 | NA |  |  |
| 17 | Primary | 09/09/2004 | 68 | 48 | Feliz Natal/MT |
|  | Relapse | 11/10/2004 | 2004 |  |  |
|  | Relapse | 20/11/2004 | 1260 |  |  |
| 18 | Primary | 06/06/2005 | NA | 42 | Ariquemes/RO |
|  | Relapse | 11/08/2005 | 1918 |  |  |
| 19 | Primary | 13/10/2005 | 5535 | 19 | Rio Branco/AC |
|  | Relapse | 14/11/2005 | 2631 |  |  |
| 20 | Primary | 25/10/2004 | 815 | 46 | Alto Paraíso/RO |
|  | Relapse | 04/01/2005 | 2810 |  |  |
| 21* | Primary | 14/11/2005 | 868 | 25 | Juruena/MT |
|  | Relapse | 29/12/2005 | 938 |  |  |
| 22* | Primary | 17/11/2004 | 1260 | 24 | Novo Progresso/PA |
|  | Relapse | 14/01/2005 | 1680 |  |  |
| 23 | Primary | 22/06/2005 | 2714 | 36 | Machadinho D'Oeste/RO |
|  | Relapse | 13/09/2005 | NA |  |  |
| 24 | Primary | 26/02/2008 | 2415 | 42 | Cruzeiro do Sul/AC |
|  | Relapse | 14/05/2008 | 1318 |  |  |
| 25* | Primary | 26/07/2005 | 593 | 63 | Humaitá/AM |
|  | Relapse | 27/01/2006 | 2680 |  |  |
| 26 | Primary | 31/07/2001 | 1142 | 25 | Porto Velho/RO |
|  | Relapse | 21/10/2001 | 5740 |  |  |
| 27* | Primary | 24/01/2005 | 675 | 63 | Colniza/MT |
|  | Relapse | 09/03/2005 | 15 |  |  |
| 28 | Primary | 04/07/2005 | 555 | 37 | Rio Machado/RO |
|  | Relapse | 03/10/2005 | 5106 |  |  |
| 29 | Primary | 24/03/2008 | 1223 | 36 | Várzea Grande/MT |
|  | Relapse | 20/05/2008 | 468 |  |  |
| 30 | Primary | 14/01/2008 | 1698 | 42 | Novo Progresso/PA |
|  | Relapse | 14/02/2008 | 3610 |  |  |
| * Primary infections correspondent to the first malaria infection of the individual’s life. | | | | | |
