# Supplementary material for: Multiple-Clone Activation of Hypnozoites Is the Leading Cause of Relapse in Plasmodium vivax Infection
Source: PLoS One. 2012 Nov 21;7(11):e49871. doi: 10.1371/journal.pone.0049871 (PMC3503861; doi:10.1371/journal.pone.0049871)
Supplement: Table S2 — Characteristics of predominant alleles from genotyping of 10 molecular markers of Plasmodium vivax infected patients. (DOCX) [file pone.0049871.s004.docx]

**Table S2. Charactheristics of predominant alleles from genotyping of 10 molecular markers of *Plasmodium vivax* infected patients**.

^a^Fragment size in base pairs (repeat unit and number of repeats); ^b^INDELS-Only fragment sizes in base pairs; ^c^Haplotypes obtained from predominant alleles combination; *Fragment with sequence deletion; NA – not available.
